# A Plasma Survey Using 38 PfEMP1 Domains Reveals Frequent Recognition of the *Plasmodium falciparum* Antigen VAR2CSA among Young Tanzanian Children

**DOI:** 10.1371/journal.pone.0031011

**Published:** 2012-01-25

**Authors:** Andrew V. Oleinikov, Valentina V. Voronkova, Isaac Tyler Frye, Emily Amos, Robert Morrison, Michal Fried, Patrick E. Duffy

**Affiliations:** 1 Seattle Biomedical Research Institute, Seattle, Washington, United States of America; 2 Department of Global Health, University of Washington, Seattle, Washington, United States of America; 3 Laboratory of Malaria Immunology and Vaccinology, National Institute of Allergy and Infectious Diseases/NIH, Rockville, Maryland, United States of America; Université Pierre et Marie Curie, France

## Abstract

PfEMP1 proteins comprise a family of variant antigens that appear on the surface of *P. falciparum*-infected erythrocytes and bind to multiple host receptors. Using a mammalian expression system and BioPlex technology, we developed an array of 24 protein constructs representing 38 PfEMP1 domains for high throughput analyses of receptor binding as well as total and functional antibody responses. We analyzed the reactivity of 561 plasma samples from 378 young Tanzanian children followed up to maximum 192 weeks of life in a longitudinal birth cohort. Surprisingly, reactivity to the DBL5 domain of VAR2CSA, a pregnancy malaria vaccine candidate, was most common, and the prevalence of reactivity was stable throughout early childhood. Reactivity to all other PfEMP1 constructs increased with age. Antibodies to the DBL2βC2**_PF11_0521_** domain, measured as plasma reactivity or plasma inhibition of ICAM1 binding, predicted reduced risk of hospitalization for severe or moderately severe malaria. These data suggest a role for VAR2CSA in childhood malaria and implicate DBL2βC2**_PF11_0521_** in protective immunity.

## Introduction

Severe malaria syndromes caused by *Plasmodium falciparum* kill over 1 million African children each year. *P. falciparum*-infected erythrocytes (IE) adhere to host endothelium and to red blood cells, allowing IE to sequester in deep vascular beds of various organs [1]–[5]. Sequestration may be related to severe complications, like cerebral malaria, placental malaria, respiratory distress, and severe anemia [6]–[10]. Parasite adhesion and sequestration are thought to be mediated by the PfEMP1 family (∼60 members per genome) of clonally variant surface-expressed erythrocyte membrane proteins [11]–[13].

The role of specific PfEMP1 proteins in severe malaria syndromes remains unclear. A single member of the PfEMP1 family, VAR2CSA, is upregulated by IE that sequester in the placenta during pregnancy malaria (PM) (reviewed in [14]). PM risk decreases after one or two pregnancies as women acquire anti-adhesion antibodies to placental parasites [15] and greater plasma reactivity to VAR2CSA [16], [17]. By comparison, severe syndromes (cerebral malaria, respiratory distress, and severe anemia) may occur only once or twice in exposed children [18], [19], and only a small number (1–2%) of parasitemia episodes progress to severe malaria [20]. This epidemiology supports a model in which severe malaria is caused by a limited number of parasite variants, possibly expressing particular PfEMP1 alleles that determine specific parasite adhesion, similar to pregnancy malaria. The rapid acquisition of resistance to severe malaria [19], [21] also implies that the targets of the protective immune response have conserved features.

Using a mammalian expression system and BioPlex technology, we developed a high throughput system to study arrays of correctly folded PfEMP1 domains for analyses of receptor binding, and for measurements of total (plasma reactivity) and functional (inhibition of receptor binding) antibody levels [22]. In previous work, using a small set of sera, we found that antibodies that inhibit binding of ICAM1 to DBL2βC2**_PF11_0521_** are common in adults and rare in infants. In the current work we expand the panel of domains to study the development of anti-PfEMP1 immunity in young children. PfEMP1 proteins belonging to group A (previously implicated in severe malaria [23]), groups B, B/C, and C (that typically bind the glycoprotein CD36), and VAR2CSA (previously implicated in pregnancy malaria [16], [17]) were included in the array as single or tandem domains. In total, we assayed 561 plasma samples from 378 young children followed up to age 192 weeks in longitudinal birth cohort studies in Tanzania [24] for reactivity to 24 protein constructs representing 38 PfEMP1 domains. We observed widespread and persistent recognition of the pregnancy malaria antigen VAR2CSA by plasma of young children. In addition, antibodies to an ICAM1-binding PfEMP1 domain, DBL2βC2**_PF11_0521_**, predicted a reduced risk of hospitalization for severe or moderately severe malaria.

## Results and Discussion

### Multi-domain NTS-DBL1-CIDR1 constructs bind CD36

In their native state, proteins exist in a folded form, which can create conformationally dependent epitopes and also mask cryptic epitopes. For this reason, correctly folded recombinant proteins may have lower seroreactivity than unfolded proteins [17], [25], which could be explained by cryptic epitopes. Consequently, the use of correctly folded proteins for seroreactivity studies might yield clearer associations with clinical outcomes by displaying relevant epitopes, but not irrelevant cryptic epitopes against which antibodies cannot bind in the native protein.

**Figure 1:**
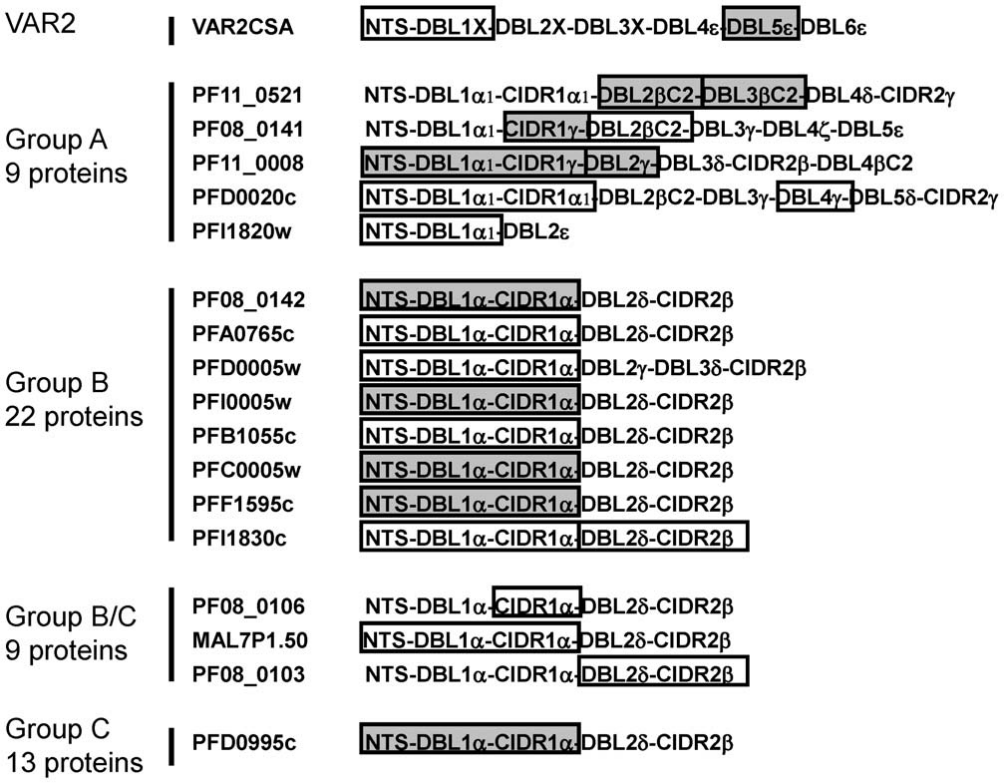
PfEMP1 domains tested for IgG reactivity using Tanzanian children's plasma. PfEMP1 domain constructs studied in this work are indicated by boxes. Shaded boxes indicate constructs recognized by ≥10% of children. *Var* gene groups according to (25) and total numbers of PfEMP1 genes from parasite clone 3D7 genome in these groups are shown on the left.

We previously expressed all DBLβC2 domains encoded in the genome of parasite clone 3D7, and surveyed their binding properties [22]. Because CIDR domains always follow DBL domains, DBL-CIDR tandems may organize structural-functional units within PfEMP1 proteins. Therefore, we prepared 14 multidomain constructs containing DBL-CIDR tandems (Figure 1), including 12 comprised of N-terminal segment (NTS)-DBL1-CIDR1 domains that constitute the semi-conserved PfEMP1 N-terminal head structure [26]. These head structures, as well as 3 single CIDR1 domains and 2 NTS-DBL1 constructs, were prepared and tested for binding to CD36, as we previously described for DBLβC2::ICAM1 interactions [22].

The CD36 binding results obtained with multidomain constructs that contain CIDR1 (Table 1) correspond qualitatively to results reported earlier for single CIDR1 domains in a different system [26]. Based on dilution curves, CIDR1-containing constructs of PFD0995c, PFI0005w, PFC0005w, PFD0005w, and PFF1595 bind CD36 with higher affinity than other variants, and PF08_0106 binds with very high affinity (Table 1). As expected [26], individual DBL1 domains (n = 2) fail to bind CD36. Thus, the domain boundaries and expression platform allow these complex multidomain polypeptides to correctly fold into functional units.

**Table 1:** Levels of CD36 binding to N-terminal head structures (NTS-DBL1-CIDR1) or their CIDR1 domains (CIDR1).

Group	Gene	Domain	Binding (AU±SD) to CD36 (µg/ml)		
			5	1	0.25
A	PF08_0141	CIDR1γ	0	0	0
A	PF11_0008	NTS-DBL1α1 -CIDR1γ*	0	0	0
A	PFD0020c	NTS-DBL1α1-CIDR1α1*	0	0	0
A	PFI1820w	NTS-DBL1α1*	0	0	0
B	PF08_0142	NTS-DBL1α-CIDR1α	2255±268	198±4	36±25
B	PFA0765c	NTS-DBL1α-CIDR1α	2505±119	319±5	85±26
B	PFD0005w	NTS-DBL1α-CIDR1α	2442±64	543±117	149±9
B	PFI0005w	NTS-DBL1α-CIDR1α	2544±309	1727±890	0
B	PFB1055c	NTS-DBL1α-CIDR1α	2732±206	0	0
B	PFC0005w	NTS-DBL1α-CIDR1α	2740±81	1338±734	NT
B	PFF1595c	NTS-DBL1α-CIDR1α	2780±230	921±190	NT
B	PFI1830c	NTS-DBL1α-CIDR1α	2775±272	0	0
B/C	PF08_0106	NTS-DBL1α	0	0	0
B/C	PF08_0106	CIDR1α	13961±7	11930±228	6594±64
B/C	MAL7P1.50	NTS-DBL1α-CIDR1α	NT	2327±464	NT
C	PFD0995c	NTS-DBL1α-CIDR1α	2490±115	1740±939	339±35
C	PFD0995c	CIDR1α*	3456±682	2717±653	NT

*Domains cloned into pAdEx, other domains cloned into pHisAdEx.

Abbreviations: AU, Arbitrary Units; SD, Standard Deviation; NT, Not Tested.

In an earlier study, seroreactivity in African children was acquired most rapidly against group A or A/B DBL1 domains, but conversely was acquired most rapidly against group B, B/C, or C CIDR1 domains [27]. This inconsistency has been attributed to higher cross-reactivity against CIDR domains versus DBL domains [28]. However only two CIDR1 domains from group B have been tested and showed minimal cross-reactivity with group A domains [28], and therefore more data are needed to resolve this issue. The DBL-CIDR tandem constructs included in the present study avoid any domain-specific bias in analyses of the plasma reactivity of PfEMP1 head structures, and may therefore be superior targets for seroreactivity studies.

### Plasma reactivity against VAR2CSA PfEMP1 protein is common in infants

**Figure 2:**
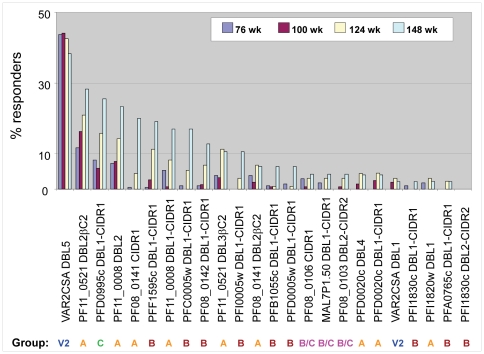
Prevalence of plasma IgG reactivity to individual PfEMP1 domains, stratified by age. Bars indicate the prevalence of plasma IgG reactivity among Tanzanian children of different ages to the indicated PfEMP1 constructs. PfEMP1 classification: V2 - VAR2CSA, A - group A, B - group B, C - group C, B/C - group B/C (according to [29]). As indicated in Figure 1 and Table 1, DBL1-containing constructs also contain N-terminal segment (NTS), but NTS is not included in the construct names.

Eleven out of 24 domain constructs were recognized by >10% of serum samples from children above 2 years of age (Figure 1, and Figure 2). These included 5 out of 9 PfEMP1 Group A constructs, 5 out of 13 domains from groups B, B/C, and C, and 1 out of the 2 VAR2CSA constructs (based on the proposed *var* gene classification [29]). At age 76 weeks, antibody prevalence is highest against VAR2CSA DBL5 and group A domains (Figure S1 A and B), while at later ages the prevalence of reactivity to groups B&B/C domains increases disproportionately. Group A *var* gene expression has been associated with severe malaria in some studies [23], [30]–[32], and our data are consistent with the hypothesis that parasites expressing group A PfEMP1 proteins prevail in the youngest children [27] and this might contribute to severe malaria. Alternatively, the pattern of plasma reactivity might also be due to greater immunogenicity or higher sequence conservation of Group A versus other *var* genes.

Antibodies to specific non-Group A PfEMP1 proteins that bind avidly to CD36 (for example, PFD0995c, PFF1595c, PF08_0106, Table 1) arose as quickly as those to Group A proteins. Similarly, DBL2βC2**_PF11_0521_**, which binds strongly to ICAM1, was the second most commonly recognized PfEMP1 domain after VAR2CSA. In general, antibodies against ICAM1-binding domain dominate at early ages, while antibodies against CD36-binding domains increase at later ages (Figure S1 A and B). These data support the notion that parasites expressing highly adhesive PfEMP1 proteins may have a growth advantage and cause disease in non-immune young children [27], but quickly induce specific antibodies that control these parasites and reduce future disease risk. If so, a fuller understanding of PfEMP1 binding properties will be critical to develop preventive or therapeutic interventions, and can be facilitated by our high throughput assay for PfEMP1 domains.

The prevalence of reactivity increased with age to all PfEMP1 proteins recognized by more than 5% of children except VAR2CSA. The DBL5 domain of VAR2CSA was the best-recognized antigen, and the prevalence of reactivity to this domain was stable throughout early life (Figure 2). The DBL1 domain of VAR2CSA was recognized less frequently than the DBL5 domain. A similar phenomenon has been observed for other PfEMP1 proteins: 55% of Tanzanian children recognize the MAL6P1.4 DBL5 domain versus only 1% that recognize the MAL6P1.4 DBL3 domain [27]. These differences have been ascribed to rare combinations of the discordant domains within one protein but might also be due to differences in immunogenicity, surface exposure, or sequence variability of the domains within the same protein. The DBL4 domain in VAR2CSA PfEMP1 protein, a main target of pregnancy malaria vaccine [16], [17], [33], clearly demonstrates discordant induction of antibody. Plasma from multigravid women infrequently react to this domain (26% responders; zero median reactivity) compared to VAR2CSA DBL5 and DBL3 domains (82% responders to each and significant positive median reactivity) [17], even though these 3 domains are shared by all VAR2CSA alleles sequenced to date.

The magnitude of the immune response against DBL5 ranged from 0 to 6674 AU among children (Figure S2A), with a maximum comparable to that of pooled plasma of African multigravidae at 11093 AU. Despite the putative role of VAR2CSA in PM, more than a third of children at any age demonstrated reactivity to this domain (Figure 2), and more than half of children had at least one of their samples react to it (Figure S2B). Pregnancy malaria, measured at the time of delivery for each mother by the presence of parasites in the placenta [24], did not predict subsequent plasma reactivity to VAR2CSA in these children (p = 0.43, Fisher's exact test) or correlate with anti-DBL5 IgG levels (Spearman r = 0.078, p = 0.07).

The higher conservation of VAR2CSA sequence compared to other PfEMP1 may contribute to the greater frequency of recognition, but the sustained reactivity suggests that exposure to VAR2CSA-expressing parasites is probably common throughout early childhood. This possibility is also supported by our earlier mass-spectrometry analyses that detected VAR2CSA peptides in 4 out of 24 (17%) children's parasites [34], despite the relatively low sensitivity of mass-spectrometry to identify variant proteins. Exposure to VAR2CSA during childhood apparently does not lead to protection against pregnancy malaria in adult women, who are highly susceptible to malaria at the time of their first pregnancies.

The frequency of plasma reactivity to VAR2CSA DBL5 among children is similar to that among adult males in our East African cohort [17] and in Ghana [16], and among primigravid women in Africa [17]. The prevalence of plasma reactivity to CSA-binding parasites among adult males living in Kenya and in Papua New Guinea [35] is also comparable (35–50% responders, depending on strain). Moreover, the same study [35] reports that at least 13% of children recognize CSA-binding CS2 parasites. Although the level and prevalence of reactivity to VAR2CSA is significantly higher in pregnant multigravid women [16], [17], the widespread reactivity in plasma from children, primigravidae, and adult males emphasizes our incomplete understanding of the protective immune response during PM. Possibly, VAR2CSA in children's parasites elicits different specificities than VAR2CSA in maternal parasites, and this might result in different proportions of non-functional and functional antibodies. For example, VAR2CSA may form complexes with different proteins, or appear on the surface at different densities, leading to different conformations or epitopes seen by the immune system. Studies by others [36] have shown that trafficking of PfEMP1 to the host cell surface is inefficient, with abundant intracellular PfEMP1 protein whose conformation and immunogenicity may differ from that of surface-displayed PfEMP1. This emphasizes the importance of studying functional immune responses, such as those that inhibit adhesion of IE surface proteins to host receptors [22].

### Anti-PfEMP1 domain plasma reactivity is dynamic

**Figure 3:**
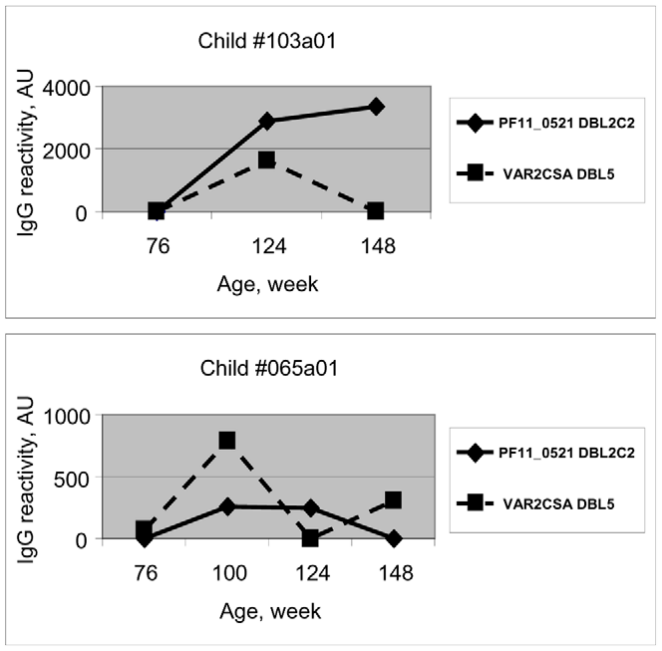
Plasma IgG reactivity to PfEMP1 domains is dynamic over time in individual children. The two panels portray two children as representative examples who experience increases and decreases in plasma reactivity over time against two commonly recognized PfEMP1 domains.

At the population level, the number of reactive PfEMP1 domains (Spearman correlation coefficient r = 0.12, P = 0.0058) and the sum of anti-PfEMP1 IgG reactivities (r = 0.12, P = 0.0033) positively correlate with the number of preceding parasitemia episodes (Figure S3). The relatively low degree of correlation may be due to the incomplete repertoire of PfEMP1 domains included in this study, and an expanded set of domains might strengthen the relationship. Plasma reactivity to individual PfEMP1 domains waxes and wanes with time in individual children (Figure 3). An increase in IgG reactivity did not always correspond to a preceding parasitemia event, even though blood smears were examined every 2–4 weeks in these children. We cannot exclude the possibility that asymptomatic parasitemia episodes occurred between monthly or bi-weekly blood smears, and may have affected plasma reactivity.

### IgG to DBL2βC2_PF11_0521_ domain predicts reduced risk of malaria hospitalizations

Infants rarely develop functional IgG [22] or plasma reactivity [22], [27] to PfEMP1 domains. In our cohort, the prevalence of reactivity increases from age 76 weeks on, and in parallel the proportion of children subsequently experiencing severe or moderately severe malaria declined sharply in the 1–2 year age window (Duffy et al., manuscript in preparation; Figure S4). For this reason, we examined the effect of antibody measured at 76 weeks of age on subsequent risk of disease. We focused our detailed analysis on the 2 domains (DBL2C2**_PF11_0521_** and VAR2CSA DBL5) that were recognized most commonly. In this cohort, eighteen children were hospitalized after age 76 weeks with severe or moderately severe malaria, and these included children who suffered severe anemia (n = 1), respiratory distress (n = 7), prostration (n = 4), convulsions (n = 2), hyperpyrexia (n = 9), or hypoglycemia (n = 1). Hyperparasitemia without other features of serious disease did not meet our criteria for severe or moderately severe malaria.

We considered two measures of plasma IgG: plasma reactivity by ELISA, and functional antibody measured as the ability of children's plasma to inhibit ICAM1 binding to the DBL2C2**_PF11_0521_** domain [22]. The amounts of total and functional IgG for the entire set of samples correlate modestly (Spearman r = 0.19, p = 0.0054), suggesting that neither assay is fully sensitive for detecting antibody responses. In contrast, the correlation between these measurements in samples from immune adults [22] is significantly stronger (r = 0.61, p = 0.0001). None of the children found to have DBL2C2**_PF11_0521_**-reactive IgG, and none of the children found to have functional antibody by binding-inhibition assay, subsequently suffered severe or moderately severe malaria. Because both assays have incomplete sensitivity, and because plasma IgG reactivity and inhibition of ICAM1 binding represent two facets of the specific anti-domain immune response that can contribute to protection, we analyzed whether responses detected by either assay would predict reduced risk of disease. Children with antibody measured in either format demonstrated a reduced risk of Severe/Moderately severe malaria hospitalizations (p = 0.048, Fisher's exact test; Table 2).

**Table 2:** Number of children hospitalized with subsequent severe or moderately severe malaria, stratified by Positive plasma reactivity (IgG>0 or BI>0) against DBL2βC2_PF11_0521_ domain measured at 76 weeks of age.

	Subsequent severe or moderately severe malaria		
	YES	NO	
**Positive Plasma Reactivity**	0	38	38
**No Plasma Reactivity**	18	166	184
	18	204	222

Fisher's Exact Text: p = 0.048, OR = 0.117 [95% CI = 0.007 to 1.98].

Please, note that the P value computed from Fisher's test is exactly correct. However, the confidence intervals for the Odds ratio (OR) are computed by methods that are only approximately correct. Because one cell contains “0”, a value of 0.5 was automatically added to each cell for calculation of OR (GraphPad Prizm software). Therefore, the confidence interval does not quite agree with the P value.

Potential confounding factors did not explain the relationship between plasma IgG and malaria hospitalization risk. We analyzed the following factors for confounding effects: 1) time from the most recent parasitemia (i.e., confounding due to acute antibody response); 2) duration of follow-up (i.e., confounding due to differential observation time); 3) number of previous parasitemia episodes (i.e., confounding due to cumulative exposure). Time from the most recent parasitemia (p = 0.36), duration of follow-up (p = 0.76), and the number of previous parasitemia episodes (p = 0.68) did not differ significantly (Mann-Whitney test) between children with versus those without DBL2C2**_PF11_0521_** specific antibodies measured in one or the other assay (Figure S5). Further, IgG directed against VAR2CSA DBL5 (p = 0.62) or any domain other than DBL2C2**_PF11_0521_**, was not associated with protection against severe or moderately severe malaria hospitalizations (Figure S6), suggesting the relationship to protection is specific to the DBL2C2**_PF11_0521_** response.

In an earlier study, IgG reactivity against the PF11_0008 CIDR2 domain was associated with protection against clinical malaria among children 4–9 years of age, but not among younger children, and plasma reactivity to the DBL1 and DBL2 domains of the same protein were not related to risk. [37]. In our work, we detected antibodies to DBL1-CIDR1 and DBL2 constructs from this gene in children below 4 years of age, and as in the previous study did not observe an association with protection against parasitemia or against severe/moderately severe malaria. In another study [38], children with IgG reactivity to VAR4 CIDR1 domain (PFD1235w gene) were less likely to have anemia (defined as hemoglobin <11 g/dl). We did not test this domain in our study.

Seroreactivity studies using variant antigens are limited to some extent because it is not possible to represent the full array of naturally occurring sequence variants. Nevertheless, our present and previous results [22] as well as results of others [27], [37], [38] suggest that meaningful conclusions are obtained despite this limitation, possibly due to sufficient conservation/cross-reactivity of specific epitopes within the variant antigens. For example, the frequent recognition of DBL2βC2**_PF11_0521_** domain by children older than 2.5 years of age (Figure 2 and Figure S1) and by adults [22] suggests substantial conservation of its epitopes among field isolates. Immunity against severe malaria may be acquired quickly in early childhood, possibly after only 1 or 2 infections [19]. In areas of high stable transmission, severe malaria incidence declines rapidly after 1.5 years of age [18], [19], and constitutes a small percentage of malaria cases. At our study site, malaria transmission is intense [24], and severe malaria hospitalizations constitute <2% of parasitemia events in children with a median follow up to age 156 weeks. Many children in endemic areas never suffer severe malaria despite multiple infectious bites, prompting speculation that they develop immunity against severe malaria parasites while protected by maternal antibodies in early infancy [19]. Even in areas of lower transmission (<1 clinical attack per year), immunity against severe malaria is largely established by the age of five years [39]. These data suggest that parasites causing severe malaria are not diverse or their diversity is significantly restricted [19], and that recognition of these parasites and their surface antigens is acquired relatively rapidly.

Our results with 561 plasma samples and 38 PfEMP1 domains find that antibodies against an ICAM1-binding DBL2βC2**_PF11_0521_** domain predict resistance to severe/moderately severe malaria hospitalization. This domain can bind host receptor ICAM1 with high affinity, similar to the affinity of anti-ICAM1 My-13 monoclonal antibody [22]. Parasite-ICAM1 interactions have been implicated in the pathogenesis of cerebral malaria [8], [9], although the evidence for this hypothesis is inconclusive. A larger sample size is needed to assess whether cerebral disease risk is specifically associated with antibodies against the DBL2βC2**_PF11_0521_** domain, and to confirm the association to protection against severe/moderately severe malaria hospitalizations. Interestingly, the DBL1α domain encoded by PF11_0521 gene belongs to *Cys2* type [40] whose transcription has been associated with severe malaria [41].

In future studies, we will expand our sample size, as well as the number of PfEMP1 domains under study, to identify the range of domains and binding interactions related to protection. Our current data encourage us to further investigate the role of DBL2βC2**_PF11_0521_** domain in severe malaria pathogenesis and immunity.

## Materials and Methods

### Ethics Statement

Human plasma samples used in these studies were collected from East African donors under protocols approved by relevant ethical review committees. Study participants provided written informed consent before donating samples. Mothers gave a written consent for themselves and their children. Ethical clearance was obtained from Institutional Review Boards of Seattle Biomedical Research Institute and the National Medical Research Coordinating Committee in Tanzania.

### Human plasma samples

Plasma samples were drawn from donors that included adult males from Kenya [42], [43] and children from Tanzania [24]. Malaria is endemic in both these regions. Plasma from 5 randomly selected non-immune donors in the US were separated from whole blood obtained from commercial sources (Valley Biomedical) and used in a pool as a negative control [22]. We tested 561 plasma samples from children (n = 378) collected at 76 (n = 222), 100 (n = 162), 124 (n = 130), and 148 (n = 47) weeks of life. Children were observed up to 192 weeks of life (median for the last visit is 136, 148.5, 168, and 184 weeks for the groups of 76, 100, 124, and 148 week tested children, respectively).

### Hospitalizations for severe and moderately severe malaria

Severe malaria was defined by WHO criteria as parasitemia together with one or more of the following: respiratory distress (respiratory rate ≥40 with physical signs of distress); two or more observed convulsions in the past 24 hours; glucose <2.2 mmol/L; prostration; hemoglobin <5.0 g/dL. Moderately severe malaria was defined as parasitemia with symptoms that did not meet WHO criteria for severe malaria but that met one or more of the following criteria: a respiratory rate ≥40; observed convulsions in the past 24 hours; hemoglobin <6.0 g/dL; temperature ≥40°C. Children with these presentations were commonly hospitalized for parenteral therapy [19], [44].

### PfEMP1 domain array construction

Construct composition and corresponding PCR primers for 26 PfEMP1 constructs encompassing 39 DBL and CIDR domains are shown in Table S1. 3D7 genomic DNA was used for PCR amplification of PfEMP1 domain constructs. PfEMP1 domain cloning into pAdEx and pHisAdEx vectors, expression in COS7 cells, and single step purification/BioPlex bead immobilization were performed as described earlier [22]. The amount of immobilized recombinant protein was tested by anti-GFP antibody and was similar for all tested constructs (data not shown). Twenty four constructs (38 domains) were used for seroepidemiology studies (Figure 1) and 17 constructs for CD36 binding studies (Table 1).

### CD36 binding activity of N-terminal head structures

Binding of CD36 at increasing concentrations to various N-terminal head structures (Table 1) was measured as we previously described for DBLβC2::ICAM1 interactions [22]. Briefly, mixtures of domains immobilized on different bead regions (Bio-Rad) were placed into wells of HTS 96-well plates (Whatmann) in duplicates. After incubation with different concentrations (5–0.25 µg/ml) of CD36-human Fc receptor (R&D Systems, Minneapolis) beads were washed and bound CD36 was detected by anti-human IgG coupled to phycoerythrin (1∶250 dilution, Jackson ImmunoResearch). Binding of CD36 to AdEx and HisAdEx constructs without inserts [22] was used as negative control in each assay. Negative control reactivity plus 2 standard deviations was subtracted from the CD36 reactivity of each PfEMP1 domain cloned into the same vector. The resulting CD36 binding activities are presented in Table 1. Binding activities of the same domains cloned into pAdEx or pHisAdEx vector were similar. Reduction of CD36 by dithiothreitol (DTT) abrogates its binding to corresponding constructs (data not shown).

### IgG reactivity of children's plasma samples against PfEMP1 constructs

This reactivity was measured as previously described [17], [22] using a BioPlex fluorometer, and the fluorescence values obtained for detection of bound IgG was described as arbitrary units (AU) in our study. The secondary antibody for this assay (Jackson Laboratory, F(ab′)2 Fragment Donkey Anti-Human IgG (H+L) (min X Bov, Ck, Gt, GP, Sy Hms, Hrs, Ms, Rb, Rat, Shp Sr Prot)), does not recognize human IgM (see below and Figure S7) that may bind non-specifically to VAR2CSA DBL5 [45]. Final reactivity of each construct was determined by subtracting the mean plus 2 standard deviations of background reactivity from the measured fluorescence values; background reactivity was defined as the highest of either 1) reactivity to the control constructs (AdEx or HisAdEx) with the same serum, or 2) mean reactivity of pooled non-immune plasma measured in all assays [17], [22].

### Reactivity of anti-human IgG with human IgG and human IgM

To confirm by standard ELISA method that anti-human IgG does not recognize human IgM we incubated 0.1 µg of human IgG and IgM in carbonate/bicarbonate buffer in duplicates using 96-well plates. After overnight incubation at 4°C, washing, and blocking in 4% milk wells were probed with various dilutions of FITC-conjugated anti-human IgG (see above). After washing, fluorescence (in arbitrary units, AU) was measured using Fluoroscan Ascent FL fluorometer/luminometer (ThermoLab systems). Results are shown in Figure S7.

### Inhibition of ICAM1 binding to DBL2βC2_PF11_0521_ domain by human plasma

These measurements (including controls) were performed as described earlier [22]. Binding inhibition (BI) was measured relative to binding buffer. BI with non-immune plasma (defined as 0% inhibition) had standard deviation (SD) of 12.5% (16 replicate samples on 8 different HTS 96-well plates), therefore children's plasma with BI above the level of 2 SD (25%) was considered as inhibitory. Pooled immune adult male serum [22] inhibited ICAM1 binding by 95±3% in these experiments.

### Statistical analyses

Analyses of proportions (contingency tables), correlation, and Mann-Whitney tests were performed using GraphPad Prizm software (version 4.03, La Jolla, CA). Multiple regression analyses were performed using StatView for Windows (version 5.0.1, SAS Institute Inc.)

## Supporting Information

Figure S1 A. Age-dependent prevalence of plasma reactivity to PfEMP1 domains ordered by decreasing reactivity measured at week 76 of life. Note that all constructs with DBL1 domain also contain NTS domain but it is not included in the names of constructs in this Figure. B. Percent of children responding to any construct in each particular PfEMP1 group, stratified by age. The total number in each age group exceeds 100% because individual children may respond to more than one antigen.
(PPT)

Figure S2 A. IgG reactivity of VAR2CSA DBL5 with plasma from children of different age after subtraction of background reactivity (mean plus 2SD threshold). AU, arbitrary units. Reactivity of pooled multigravida plasma (positive control) was 11093 AU. Highest reactivity of child plasma was 6674. Red lines indicate mean values (all median values equal to zero). Pink and blue lines indicate 2 SD and 4 SD threshold used for analysis in Figure S2B. B. Percent of children with positive IgG reactivity (detected at any age of 76 through 148 weeks for each child) at various levels of background reactivity threshold. The threshold level of 2SD used for calculations of seroreactivity in this work is underlined.
(PPT)

Figure S3 Number of reactive PfEMP1 domains (A) and the sum of anti-PfEMP1 IgG reactivities (B) positively correlate with the number of preceding parasitemia episode. Corresponding Spearman correlation coefficients (r) and P-values are indicated. Blue line demonstrates linear regression.
(PPT)

Figure S4 Opposite trends in the percentage of children with subsequent hospitalizations for severe/moderately severe malaria (A) vs. percentage of children with total or functional seroreactivity against DBL2C2PF11_0521 domain (B) with age. Total/functional reactivity is Positive if IgG or binding inhibition activity of serum against this domain is above 0. “n” indicate actual numbers of hospitalized children (A) and children with Positive reactivity (B) in the cohort.
(PPT)

Figure S5 Analysis of potential confounding factors for groups of children with Positive (IgG>0 or BI>0) and No reactivity against DBL2C2PF11_0521 domain at week 76 of age. Red lines indicate medians. P values calculated using Mann-Whitney test.
(PPT)

Figure S6 Number of children with subsequent severe or moderately severe malaria, stratified by positive and no plasma IgG reactivity against 3 most reactive constructs measured at 76 weeks of age, and P values against all constructs obtained in similar analyses.
(DOC)

Figure S7 Reactivity of anti-human IgG with human IgG and human IgM. PBST, Phosphate-buffered saline buffer containing 0.05% Tween-20 (negative control). The preparation of anti-human IgG does not recognize human IgM.
(PPT)

Table S1 Construct composition and corresponding PCR primers for 26 PfEMP1 constructs encompassing 39 DBL and CIDR domains. Amino acid residue numbers are shown according to PlasmoDB database.
(XLS)
