# Supplementary material for: A Plasma Survey Using 38 PfEMP1 Domains Reveals Frequent Recognition of the Plasmodium falciparum Antigen VAR2CSA among Young Tanzanian Children
Source: PLoS One. 2012 Jan 25;7(1):e31011. doi: 10.1371/journal.pone.0031011 (PMC3266279; doi:10.1371/journal.pone.0031011)
Supplement: Figure S1 — A. Age-dependent prevalence of plasma reactivity to PfEMP1 domains ordered by decreasing reactivity measured at week 76 of life. Note that all constructs with DBL1 domain also contain NTS domain but it is not included in the names of constructs in this Figure. B. Percent of children responding to any construct in each particular PfEMP1 group, stratified by age. The total number in each age group exceeds 100% because individual children may respond to more than one antigen. (PPT) [file pone.0031011.s001.ppt]

## Slide 1
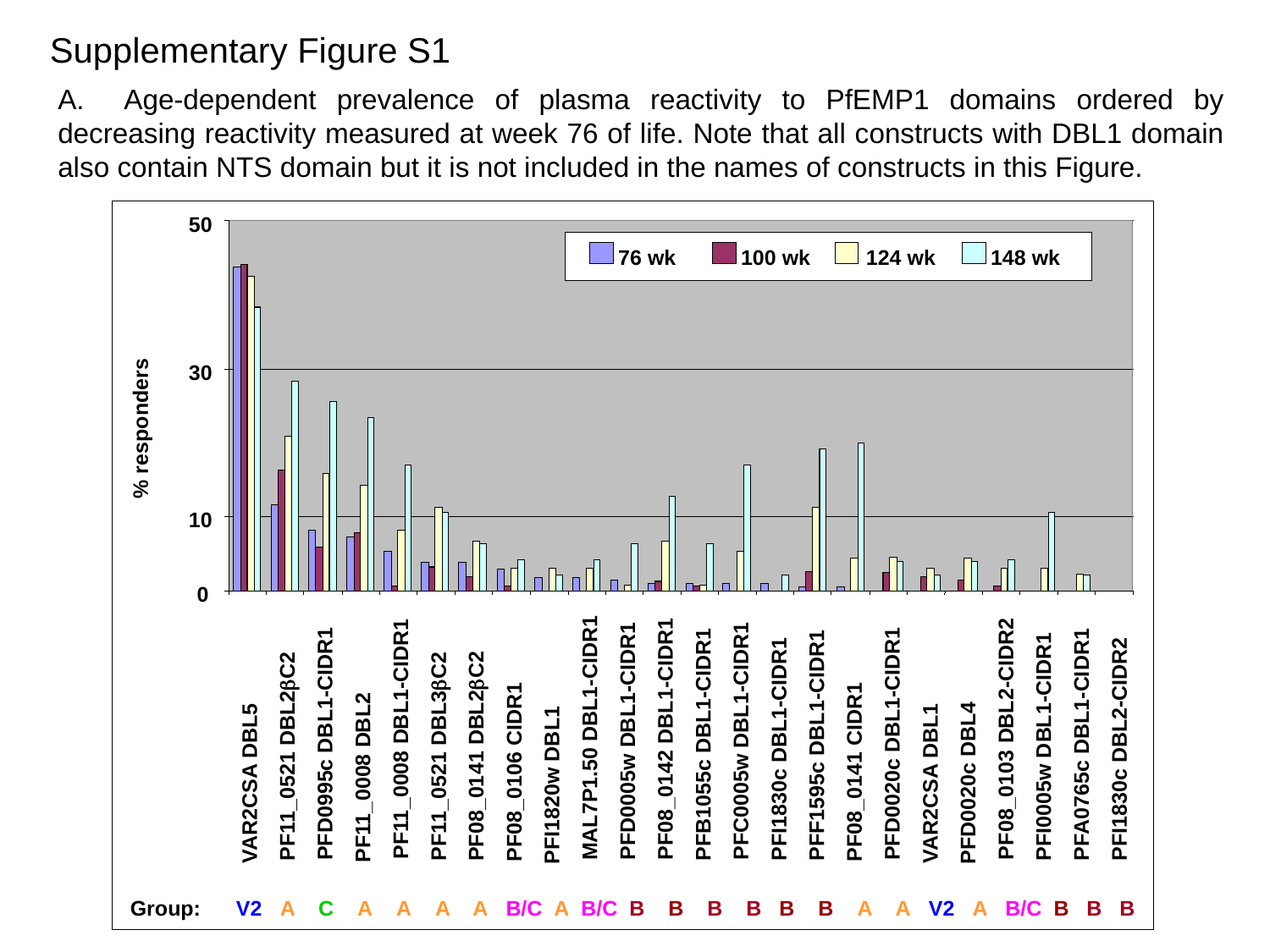

Supplementary Figure S1
A. Age-dependent prevalence of plasma reactivity to PfEMP1 domains ordered by decreasing reactivity measured at week 76 of life. Note that all constructs with DBL1 domain also contain NTS domain but it is not included in the names of constructs in this Figure.
50
76 wk
100 wk
124 wk
148 wk
30
% responders
10
0
MAL7P1.50 DBL1-CIDR1
PF11_0008 DBL1-CIDR1
PF08_0142 DBL1-CIDR1
PF08_0103 DBL2-CIDR2
PFD0005w DBL1-CIDR1
PFC0005w DBL1-CIDR1
PFD0995c DBL1-CIDR1
PFD0020c DBL1-CIDR1
PFB1055c DBL1-CIDR1
PFA0765c DBL1-CIDR1
PFF1595c DBL1-CIDR1
PFI0005w DBL1-CIDR1
PFI1830c DBL1-CIDR1
PFI1830c DBL2-CIDR2
PF11_0521 DBL2βC2
PF11_0521 DBL3βC2
PF08_0141 DBL2βC2
PF08_0106 CIDR1
PF08_0141 CIDR1
PF11_0008 DBL2
PFD0020c DBL4
VAR2CSA DBL5
VAR2CSA DBL1
PFI1820w DBL1
 Group: V2 A C A A A A B/C A B/C B B B B B B A A V2 A B/C B B B

## Slide 2
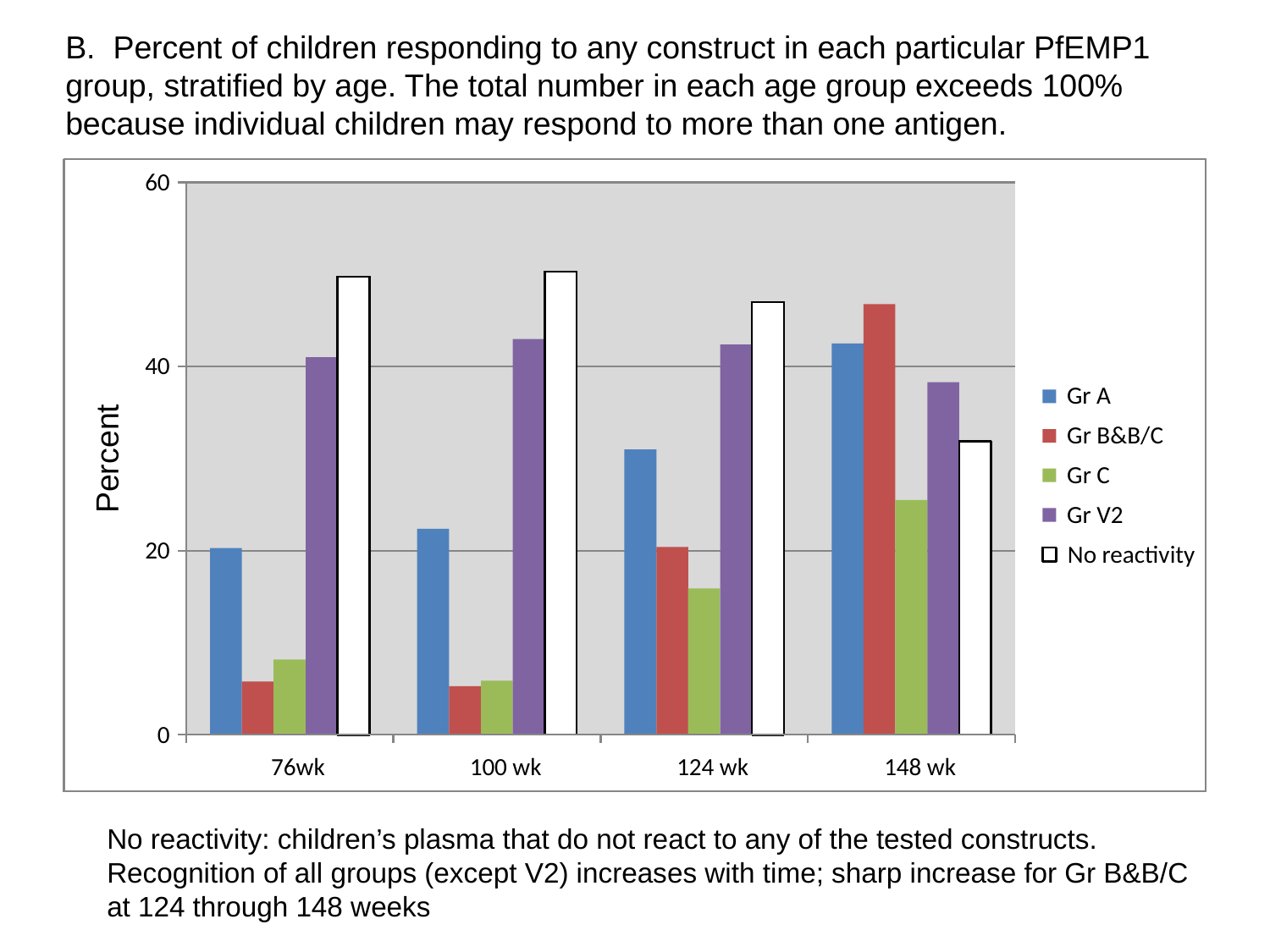

B. Percent of children responding to any construct in each particular PfEMP1 group, stratified by age. The total number in each age group exceeds 100% because individual children may respond to more than one antigen.
60
40
Gr A
Gr B&B/C
Percent
Gr C
Gr V2
20
No reactivity
0
76wk
100 wk
124 wk
148 wk
No reactivity: children’s plasma that do not react to any of the tested constructs.
Recognition of all groups (except V2) increases with time; sharp increase for Gr B&B/C at 124 through 148 weeks
