# Supplementary material for: A Plasma Survey Using 38 PfEMP1 Domains Reveals Frequent Recognition of the Plasmodium falciparum Antigen VAR2CSA among Young Tanzanian Children
Source: PLoS One. 2012 Jan 25;7(1):e31011. doi: 10.1371/journal.pone.0031011 (PMC3266279; doi:10.1371/journal.pone.0031011)
Supplement: Figure S2 — A. IgG reactivity of VAR2CSA DBL5 with plasma from children of different age after subtraction of background reactivity (mean plus 2SD threshold). AU, arbitrary units. Reactivity of pooled multigravida plasma (positive control) was 11093 AU. Highest reactivity of child plasma was 6674. Red lines indicate mean values (all median values equal to zero). Pink and blue lines indicate 2 SD and 4 SD threshold used for analysis in Figure S2B. B. Percent of children with positive IgG reactivity (detected at any age of 76 through 148 weeks for each child) at various levels of background reactivity threshold. The threshold level of 2SD used for calculations of seroreactivity in this work is underlined. (PPT) [file pone.0031011.s002.ppt]

## Slide 1
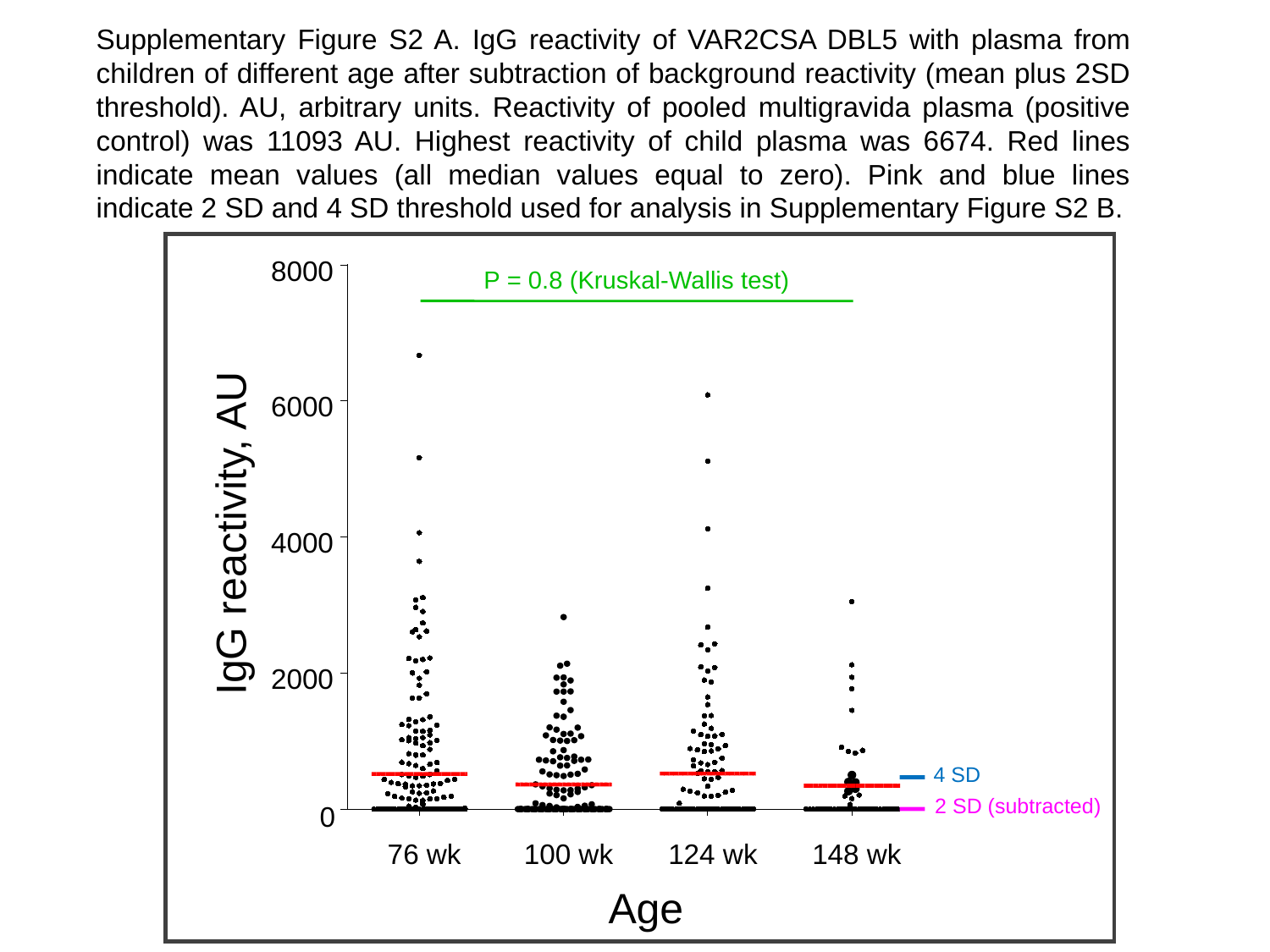

Supplementary Figure S2 A. IgG reactivity of VAR2CSA DBL5 with plasma from children of different age after subtraction of background reactivity (mean plus 2SD threshold). AU, arbitrary units. Reactivity of pooled multigravida plasma (positive control) was 11093 AU. Highest reactivity of child plasma was 6674. Red lines indicate mean values (all median values equal to zero). Pink and blue lines indicate 2 SD and 4 SD threshold used for analysis in Supplementary Figure S2 B.
8000
6000
4000
2000
0
P = 0.8 (Kruskal-Wallis test)
76 wk
100 wk
124 wk
148 wk
IgG reactivity, AU
4 SD
2 SD (subtracted)
Age

## Slide 2
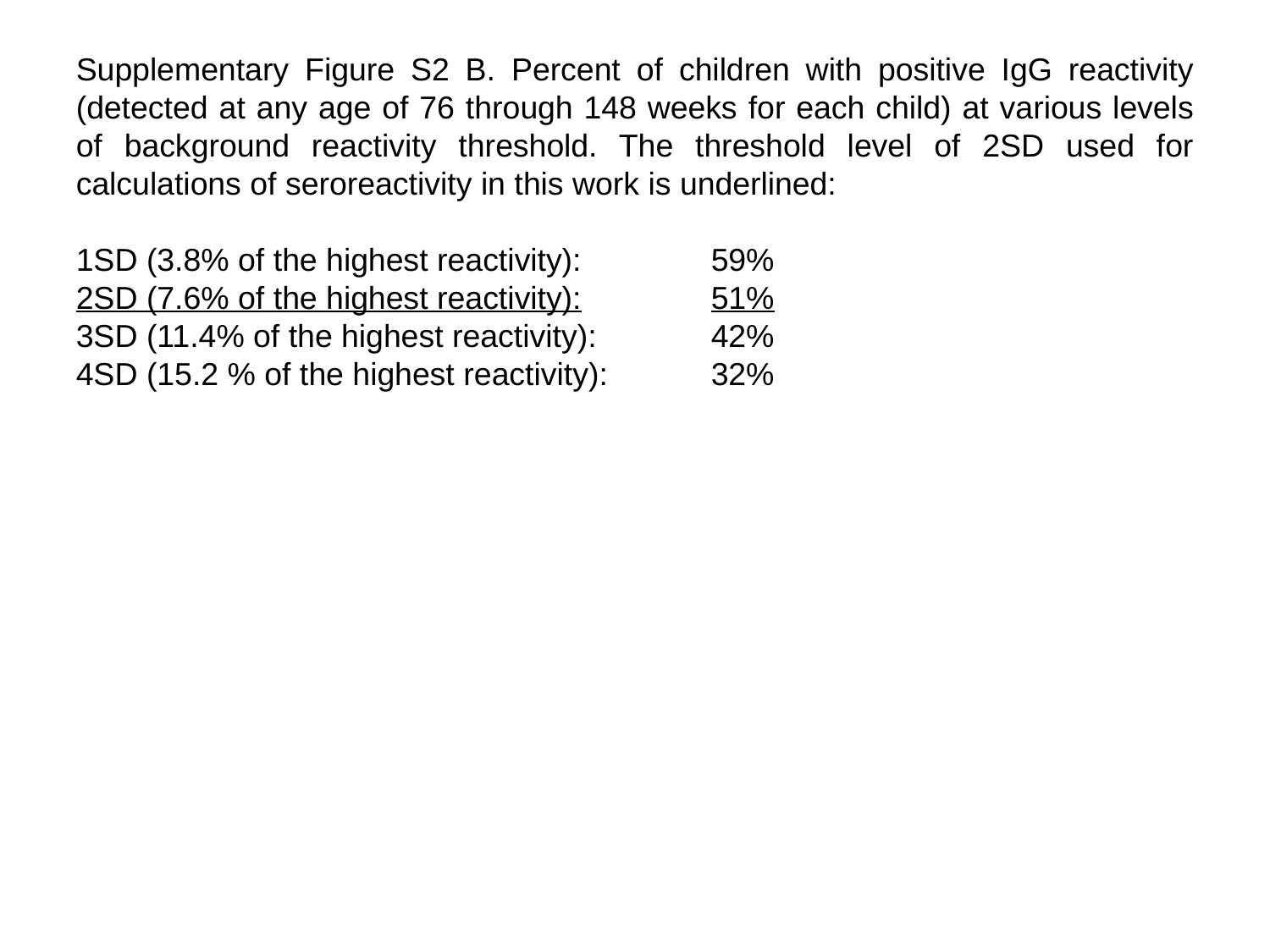

Supplementary Figure S2 B. Percent of children with positive IgG reactivity (detected at any age of 76 through 148 weeks for each child) at various levels of background reactivity threshold. The threshold level of 2SD used for calculations of seroreactivity in this work is underlined:
1SD (3.8% of the highest reactivity): 	59%
2SD (7.6% of the highest reactivity):		51%
3SD (11.4% of the highest reactivity):	42%
4SD (15.2 % of the highest reactivity):	32%
