# Supplementary material for: A Plasma Survey Using 38 PfEMP1 Domains Reveals Frequent Recognition of the Plasmodium falciparum Antigen VAR2CSA among Young Tanzanian Children
Source: PLoS One. 2012 Jan 25;7(1):e31011. doi: 10.1371/journal.pone.0031011 (PMC3266279; doi:10.1371/journal.pone.0031011)
Supplement: Figure S3 — Number of reactive PfEMP1 domains (A) and the sum of anti-PfEMP1 IgG reactivities (B) positively correlate with the number of preceding parasitemia episode. Corresponding Spearman correlation coefficients (r) and P-values are indicated. Blue line demonstrates linear regression. (PPT) [file pone.0031011.s003.ppt]

## Slide 1
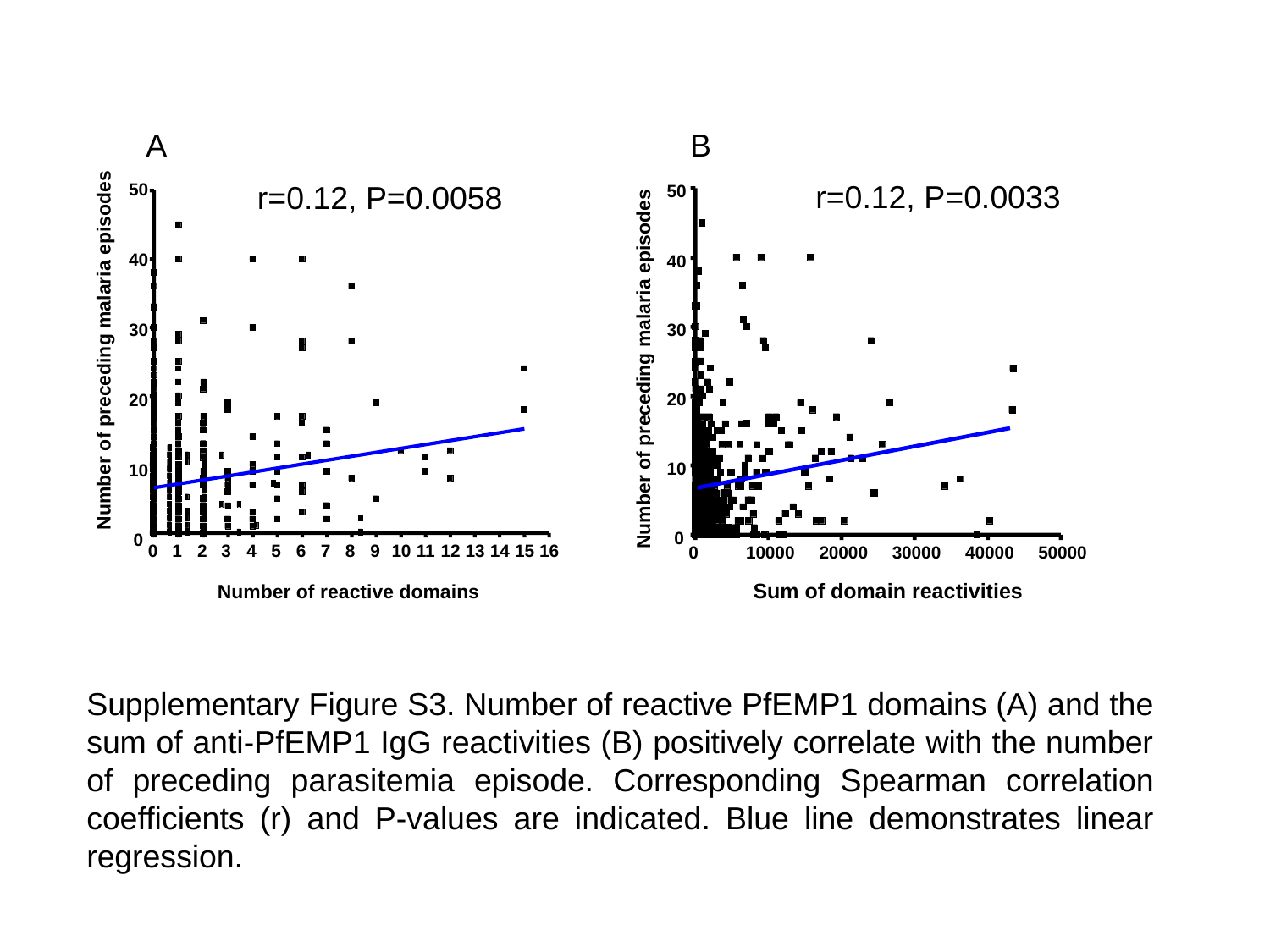

A
50
0
1
2
3
4
5
6
7
8
9
10
11
12
13
14
15
16
40
30
Number of preceding malaria episodes
20
10
0
Number of reactive domains
B
50
40
30
Number of preceding malaria episodes
20
10
0
0
10000
20000
30000
40000
50000
Sum of domains reactivities
Sum of domain reactivities
r=0.12, P=0.0033
r=0.12, P=0.0058
Supplementary Figure S3. Number of reactive PfEMP1 domains (A) and the sum of anti-PfEMP1 IgG reactivities (B) positively correlate with the number of preceding parasitemia episode. Corresponding Spearman correlation coefficients (r) and P-values are indicated. Blue line demonstrates linear regression.
