# Supplementary material for: A Plasma Survey Using 38 PfEMP1 Domains Reveals Frequent Recognition of the Plasmodium falciparum Antigen VAR2CSA among Young Tanzanian Children
Source: PLoS One. 2012 Jan 25;7(1):e31011. doi: 10.1371/journal.pone.0031011 (PMC3266279; doi:10.1371/journal.pone.0031011)
Supplement: Figure S4 — Opposite trends in the percentage of children with subsequent hospitalizations for severe/moderately severe malaria (A) vs. percentage of children with total or functional seroreactivity against DBL2C2PF11_0521 domain (B) with age. Total/functional reactivity is Positive if IgG or binding inhibition activity of serum against this domain is above 0. “n” indicate actual numbers of hospitalized children (A) and children with Positive reactivity (B) in the cohort. (PPT) [file pone.0031011.s004.ppt]

## Slide 1
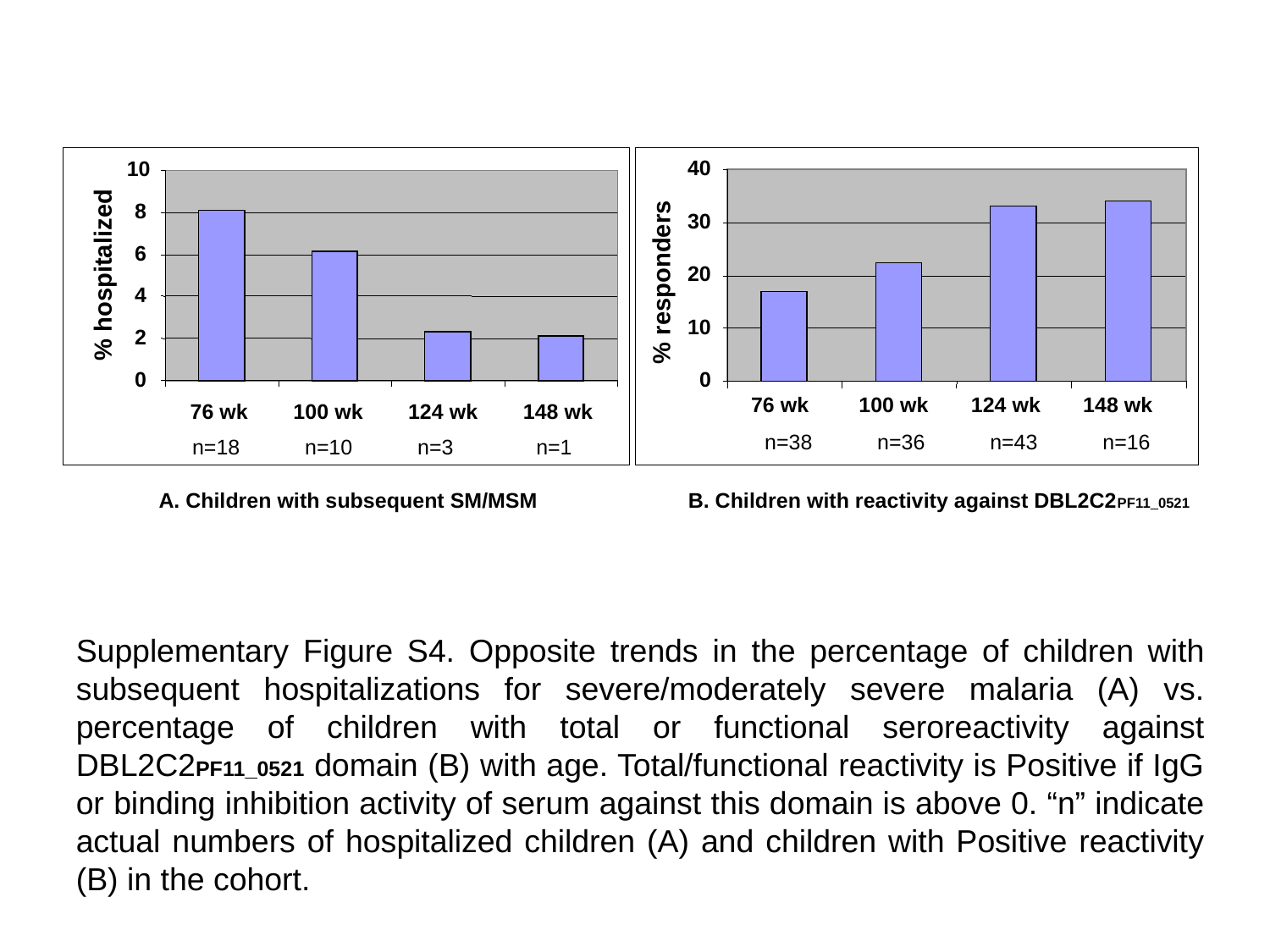

10
8
6
 % hospitalized
4
2
0
76 wk
100 wk
124 wk
148 wk
n=18 n=10 n=3 n=1
40
30
20
% responders
10
0
76 wk
100 wk
124 wk
148 wk
n=38 n=36 n=43 n=16
A. Children with subsequent SM/MSM
B. Children with reactivity against DBL2C2PF11_0521
Supplementary Figure S4. Opposite trends in the percentage of children with subsequent hospitalizations for severe/moderately severe malaria (A) vs. percentage of children with total or functional seroreactivity against DBL2C2PF11_0521 domain (B) with age. Total/functional reactivity is Positive if IgG or binding inhibition activity of serum against this domain is above 0. “n” indicate actual numbers of hospitalized children (A) and children with Positive reactivity (B) in the cohort.
