# Supplementary material for: A Plasma Survey Using 38 PfEMP1 Domains Reveals Frequent Recognition of the Plasmodium falciparum Antigen VAR2CSA among Young Tanzanian Children
Source: PLoS One. 2012 Jan 25;7(1):e31011. doi: 10.1371/journal.pone.0031011 (PMC3266279; doi:10.1371/journal.pone.0031011)
Supplement: Figure S5 — Analysis of potential confounding factors for groups of children with Positive (IgG>0 or BI>0) and No reactivity against DBL2C2PF11_0521 domain at week 76 of age. Red lines indicate medians. P values calculated using Mann-Whitney test. (PPT) [file pone.0031011.s005.ppt]

## Slide 1
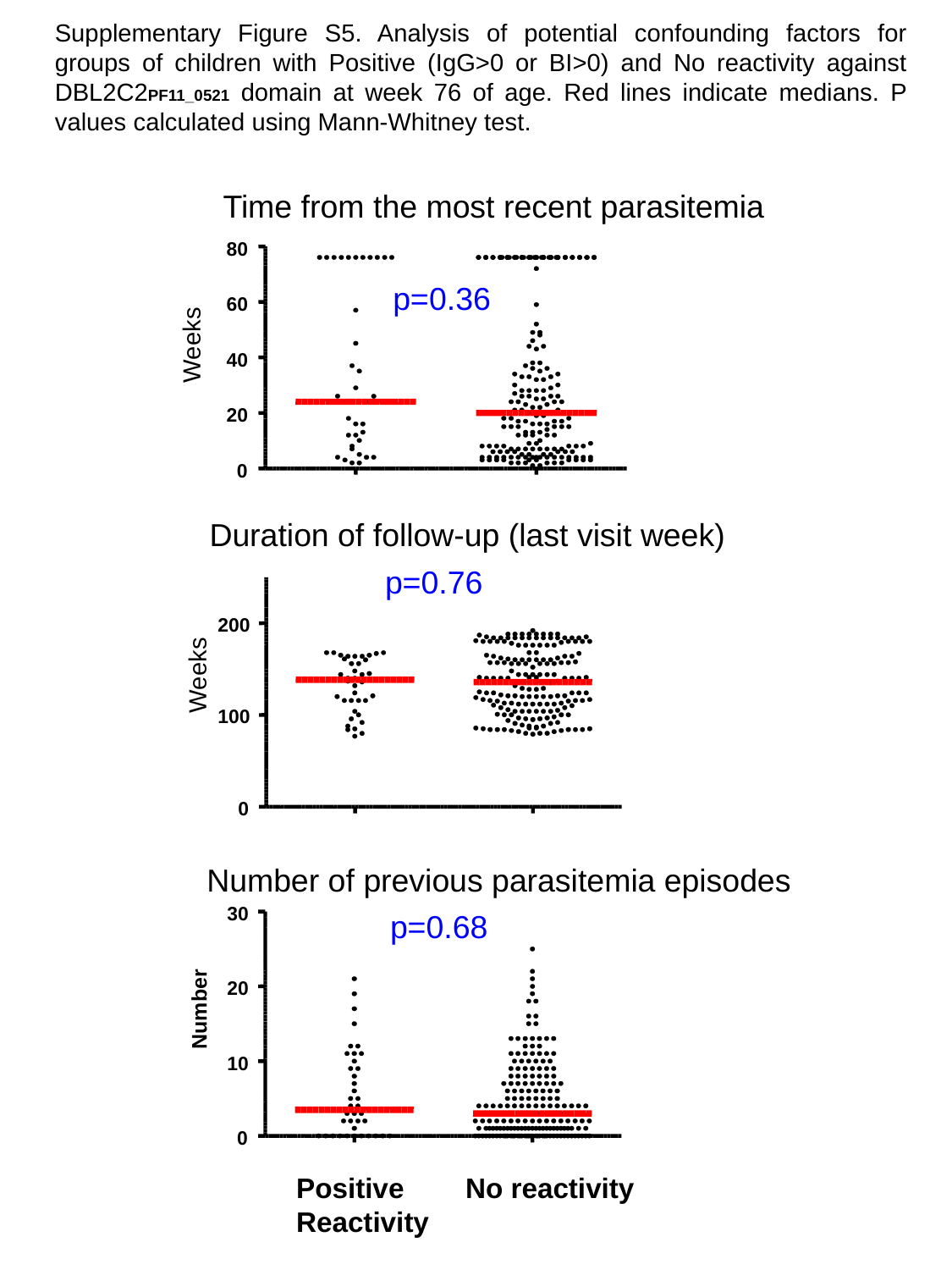

Supplementary Figure S5. Analysis of potential confounding factors for groups of children with Positive (IgG>0 or BI>0) and No reactivity against DBL2C2PF11_0521 domain at week 76 of age. Red lines indicate medians. P values calculated using Mann-Whitney test.
Time from the most recent parasitemia
80
60
40
20
0
p=0.36
Weeks
Duration of follow-up (last visit week)
p=0.76
200
Weeks
100
0
Number of previous parasitemia episodes
p=0.68
30
20
Number
10
0
Positive
Reactivity
No reactivity
