# Supplementary material for: A Plasma Survey Using 38 PfEMP1 Domains Reveals Frequent Recognition of the Plasmodium falciparum Antigen VAR2CSA among Young Tanzanian Children
Source: PLoS One. 2012 Jan 25;7(1):e31011. doi: 10.1371/journal.pone.0031011 (PMC3266279; doi:10.1371/journal.pone.0031011)
Supplement: Figure S6 — Number of children with subsequent severe or moderately severe malaria, stratified by positive and no plasma IgG reactivity against 3 most reactive constructs measured at 76 weeks of age, and P values against all constructs obtained in similar analyses. (DOC) [file pone.0031011.s006.doc]

**Supplementary Figure S6. Number of children with subsequent severe or moderately severe malaria, stratified by positive and no plasma IgG reactivity against 3 most reactive constructs measured at 76 weeks of age, and P values against all constructs obtained in similar analyses.**

**DBL5 VAR2CSA**

**
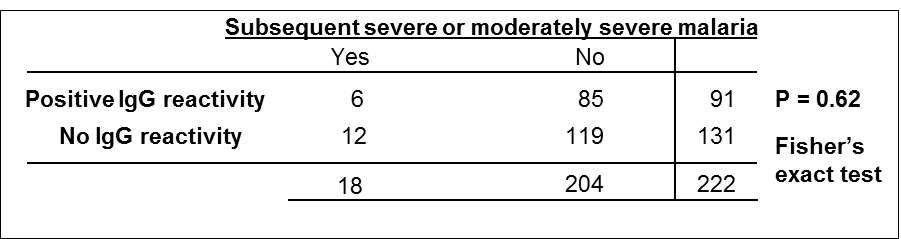
**

**NTS-DBL1-CIDR1 PFD0995c**


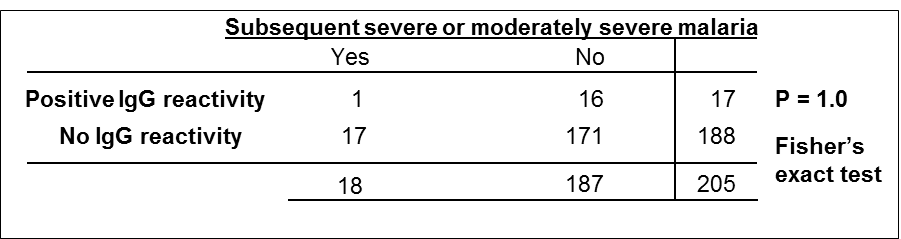


**DBL2 PF11_0008**


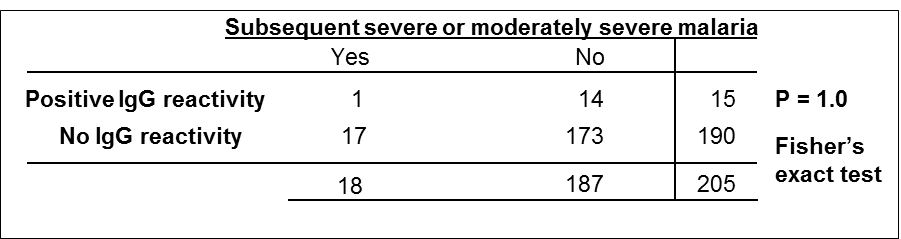


All constructs P value

VAR2CSA DBL5 0.62

PF11-0521 DBL2βC2 0.048 (Table 2); Based on IgG reactivity only p=0.13

PFD0995 NTS-DBL1-CIDR1 1.0

PF11-0008 DBL2 1.0

PF11-0008 NTS-DBL1-CIDR1 0.60

PF11-0521 DBL3βC2 1.0

PF08-0141 DBL2βC2 1.0

PF08-0106 CIDR1 1.0

PFI1820w NTS-DBL1 1.0

MAL7P1.50 NTS-DBL1-CIDR1 1.0

PFD0005 NTS-DBL1-CIDR1 1.0

PF08-0142 NTS-DBL1-CIDR1 1.0

PFB1055 NTS-DBL1-CIDR1 1.0

PFC0005 NTS-DBL1-CIDR1 1.0

PFI1830c NTS-DBL1-CIDR1 1.0

PFF1595 NTS-DBL1-CIDR1 1.0

PF08-0141 CIDR1 1.0

PFD0020c NTS-DBL1-CIDR1 1.0

VAR2CSA NTS-DBL1 N/A

PFD0020c DBL4 N/A

PF08_0103 DBL2CIDR2 N/A

PFI0005 NTS-DBL1-CIDR1 N/A

PFA0765c NTS-DBL1-CIDR1 N/A

PFI1830 DBL2CIDR2 N/A

N/A = not applicable because 2 cells in a contingency table are zeroes (no positive IgG reactivity).
