# Supplementary material for: A Plasma Survey Using 38 PfEMP1 Domains Reveals Frequent Recognition of the Plasmodium falciparum Antigen VAR2CSA among Young Tanzanian Children
Source: PLoS One. 2012 Jan 25;7(1):e31011. doi: 10.1371/journal.pone.0031011 (PMC3266279; doi:10.1371/journal.pone.0031011)
Supplement: Figure S7 — Reactivity of anti-human IgG with human IgG and human IgM. PBST, Phosphate-buffered saline buffer containing 0.05% Tween-20 (negative control). The preparation of anti-human IgG does not recognize human IgM. (PPT) [file pone.0031011.s007.ppt]

## Slide 1
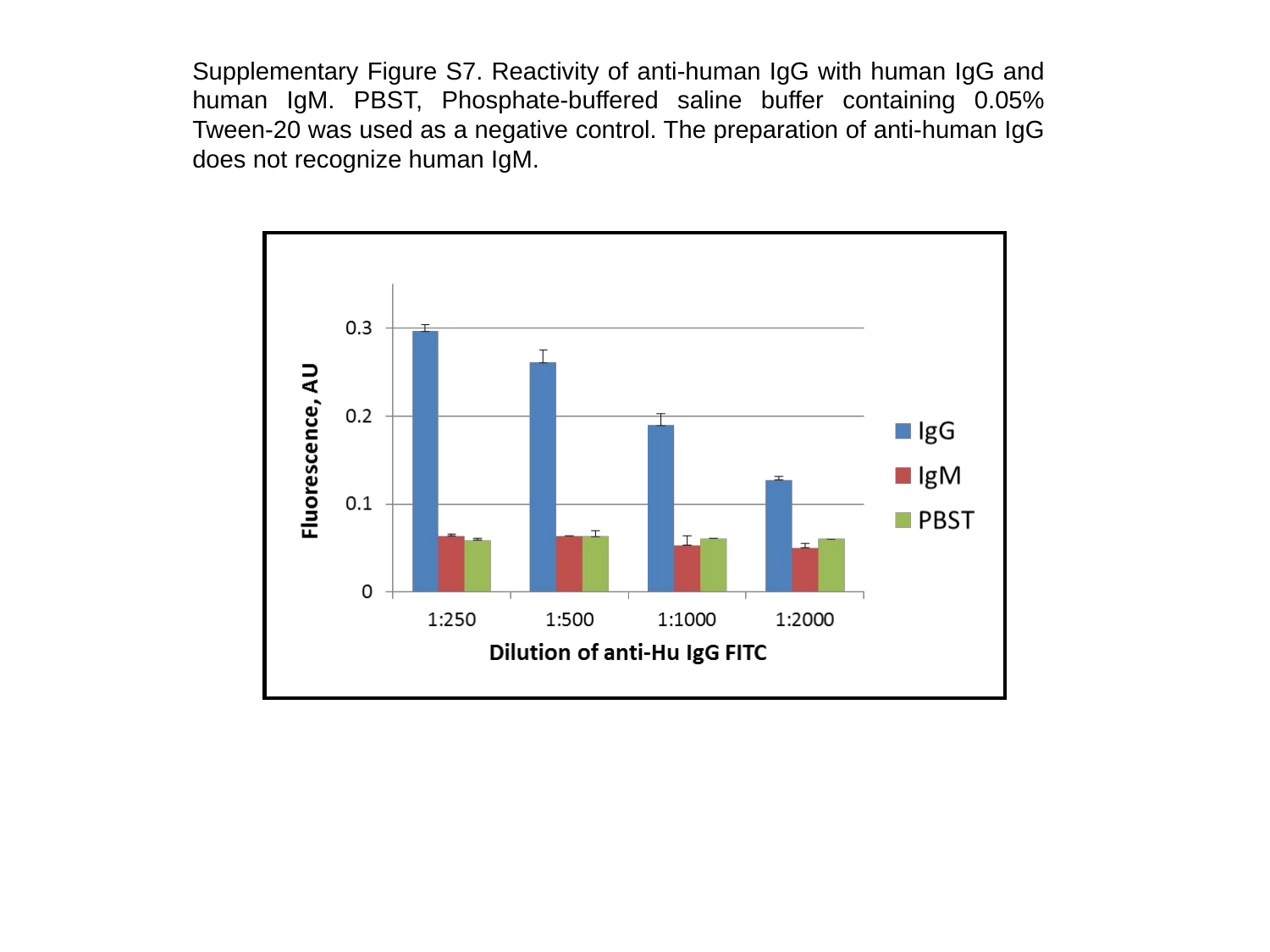

Supplementary Figure S7. Reactivity of anti-human IgG with human IgG and human IgM. PBST, Phosphate-buffered saline buffer containing 0.05% Tween-20 was used as a negative control. The preparation of anti-human IgG does not recognize human IgM.
